# Hydromorphological Restoration of Streams Does Not Ensure the Recovery of Ecosystem Functioning

**DOI:** 10.1111/gcb.71126

**Published:** 2026-10-22

**Authors:** Ulrike Haase, Daniel C. Allen, Christine Anlanger, Iola G. Boëchat, Clara Mendoza‐Lera, Wyatt F. Cross, Pia Ebeling, Davi G. Fernandes Cunha, Patrick Fink, Nicolas Finkler, Björn Gücker, Andreas Lorke, Julia Pasqualini, Christopher J. Patrick, Marc Peipoch, Oskar Schröder, Ryan Sponseller, Daniel von Schiller, Markus Weitere, Mario Brauns

**Affiliations:** ^1^ Institute of Hydrobiology Technische Universität Dresden Dresden Germany; ^2^ Department of Ecosystem Science and Management Pennsylvania State University University Park Pennsylvania USA; ^3^ RPTU University Kaiserslautern‐Landau Landau Germany; ^4^ Department of Geosciences Federal University of São João del‐Rei São João del‐Rei MG Brazil; ^5^ Department of Ecology Montana State University Bozeman Montana USA; ^6^ Department of Hydrogeology Helmholtz Centre for Environmental Research‐UFZ Leipzig Germany; ^7^ São Carlos School of Engineering University of São Paulo São Carlos Brazil; ^8^ Institute of Zoology University of Cologne Köln Germany; ^9^ Department of Forest Ecology and Management Swedish University of Agricultural Sciences Umeå Sweden; ^10^ Department of River Ecology Helmholtz Centre for Environmental Research–UFZ Magdeburg Germany; ^11^ Department of Biological Sciences Virginia Institute of Marine Science Gloucester Point Virginia USA; ^12^ Stroud Water Research Center Avondale Pennsylvania USA; ^13^ Department of Ecology, Environment and Geoscience Umeå University Umeå Sweden; ^14^ Departament de Biologia Evolutiva, Ecologia i Ciències Ambientals/Institut de Recerca de L'aigua Universitat de Barcelona Barcelona Spain

**Keywords:** biodiversity–ecosystem function relationship, denitrification, ecosystem respiration, gross primary production, meta‐analysis, nutrient uptake, organic matter decomposition, restoration, secondary production, sediment respiration

## Abstract

Ecological restoration is widely expected to recover multiple ecosystem attributes, yet biological structure and ecosystem functioning may respond differently to hydromorphological restoration. Interpreting functional change is further complicated because higher or lower process rates do not inherently indicate recovery; their ecological meaning depends on how degraded and restored conditions compare with an appropriate reference state. We synthesised 582 effect sizes from 66 studies to quantify responses of nine ecosystem functions to hydromorphological restoration of streams and to assess whether changes in biological structure coincided with functional responses. Ecosystem‐function responses varied substantially among processes and baselines. Gross primary production was significantly higher in degraded than in reference streams, illustrating that elevated functional rates do not necessarily indicate reference‐like ecological conditions. Restoration did not consistently reverse degradation‐associated functional differences: denitrification was significantly lower in restored than in degraded streams, whereas nitrate uptake remained significantly lower in restored than in reference streams. Other restored–reference differences were generally weak or uncertain. Functional responses did not differ systematically among broad restoration‐measure categories. Restoration age was associated with changes in gross primary production and denitrification relative to degraded conditions, but none of the functions showed detectable convergence towards or divergence from reference conditions through time. In studies measuring both biological structure and ecosystem functioning, biological responses to restoration were generally positive relative to degraded conditions, whereas functional responses were more variable. However, the available evidence was too uncertain to conclude that biological and functional responses systematically differed. Our synthesis shows that functional recovery cannot be inferred either from restoration‐associated change alone or from concurrent biological recovery. Assessing restoration success therefore requires direct measurements of ecosystem processes and ecologically meaningful reference conditions that reveal whether functional change represents movement towards a process‐specific restoration target.

## Graphical abstract

Hydromorphological stream restoration does not consistently restore ecosystem functioning. Across 66 studies, functional responses were heterogeneous, depended on the comparison baseline, and were not consistently aligned with responses of biological structure. Functional recovery therefore cannot be inferred from biological responses, instead requires quantification of ecosystem processes against appropriate reference conditions.

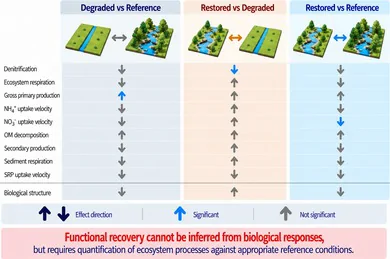

## 1 Introduction

Ecosystem restoration is expanding globally as nations commit to reversing biodiversity loss and rebuilding ecosystem services. Rivers and streams are a focal point of these efforts because they support high levels of biodiversity (Dudgeon et al. 2006), sustain key ecosystem functions such as nutrient cycling, primary production, and organic matter decomposition (von Schiller et al. 2017), and contribute a wide range of ecosystem services (Ferreira et al. 2023). In response to widespread habitat degradation and hydrological fragmentation (Schäfer et al. 2025), hydromorphological restoration has become a major approach in stream restoration (Roni et al. 2002). Measures such as instream and riparian habitat enhancement, floodplain reconnection, and barrier removal primarily aim to restore channel morphology, flow dynamics, and hydrological connectivity (Palmer et al. 2014; Wohl et al. 2015). Such interventions may affect multiple ecological attributes, including ecosystem functioning, but these responses need not occur in parallel. For example, restoring riparian vegetation may increase organic‐matter inputs and associated decomposition while reducing primary production through increased shading. Similarly, hydromorphological improvements do not necessarily translate into recovery of all stream community attributes, such as species richness, composition, or biomass (Kail et al. 2015; Roni et al. 2008). Even where biological communities respond to restoration, ecosystem functioning may not recover in parallel. Ecosystem functioning depends not only on biological structure but also on trophic interactions, microbial processes, resource availability, hydrology, nutrient supply, and other abiotic controls (Rooney and McCann 2012; Tank et al. 2010; Webster et al. 2003). Biological structure and ecosystem functioning may therefore respond differently to the same restoration intervention.

Whether biological structure and ecosystem functioning respond in parallel to restoration is closely related to questions addressed in biodiversity–ecosystem functioning (BEF) research. BEF studies have demonstrated that biodiversity can influence ecosystem processes through mechanisms such as niche complementarity and facilitative interactions (Bolger 2001; Duffy et al. 2007). However, restoration does not constitute a controlled manipulation of biodiversity. Restored stream communities are shaped by dispersal limitation, environmental filtering, historical contingency, and incomplete community reassembly (Tonkin et al. 2014), while ecosystem functions may additionally respond directly to abiotic environmental change. Consequently, positive biodiversity–function relationships established under controlled conditions do not necessarily imply that restoration‐induced changes in biological structure and ecosystem functioning will occur in parallel. Whether such responses are consistently coupled across restored stream ecosystems therefore remains unresolved.

A second major challenge is determining the baselines against which restoration outcomes are evaluated. Comparisons with degraded conditions quantify the magnitude and direction of ecological change associated with restoration, whereas comparisons with reference conditions assess how closely restored systems approach a desired ecological state (Marchand et al. 2021). This distinction is particularly important for ecosystem functions because higher functional rates do not necessarily indicate ecological improvement. Degradation can either suppress or enhance a given function depending on the dominant stressor and environmental context (Brauns et al. 2022). For example, eutrophication may elevate gross primary production above reference conditions, such that a restoration‐induced decline in gross primary production may indicate ecological recovery rather than functional loss (Hoellein et al. 2011; McTammany et al. 2007). A restored–degraded comparison therefore indicates whether an intervention changed a function, but this change can only be interpreted as recovery when the direction and magnitude of the degraded system's departure from an appropriate reference condition are known. Functional assessment thus requires process‐specific benchmarks that define realistic target ranges and the expected direction of change rather than assuming that all functions should increase following restoration.

Here, we present a global synthesis of ecosystem‐function responses and paired biological‐structure–function responses to hydromorphological restoration in stream ecosystems. Using 582 effect sizes from 66 studies, we first quantify how nine ecosystem functions respond across four broad categories of hydromorphological restoration measures, restoration ages, and comparisons among degraded, restored, and reference conditions. This baseline‐based approach allows us to distinguish restoration‐associated change from proximity to a restoration target without assuming that increases in functional rates necessarily indicate recovery. Second, we compare paired biological‐structure and functional responses to evaluate whether restoration‐induced changes in biological structure consistently coincide with changes in ecosystem functioning.

## 2 Materials and Methods

### 2.1 Literature Search and Selection

We conducted a systematic literature review in electronic databases (Web of Science and Google Scholar) for peer‐reviewed studies examining the effects of ecological restoration on ecosystem functions in running waters. The search targeted hydromorphological restoration measures and excluded chemical remediation interventions (e.g., wastewater effluent upgrades, acid mine drainage treatment), which produce fundamentally different responses and have been synthesised elsewhere (Brauns et al. 2022). The literature search was applied to titles, abstracts, and keywords using the following string: (stream OR river OR freshwater) AND (restor* OR recover* OR rehabil* OR revital*) AND (monitor OR assess* OR quantif* OR measur* OR estimat* OR evaluat*) AND (“ecosystem respiration” OR photosynthe* OR “secondary produc*” OR “primary produc*” OR “biomass produc*” OR “biomass accrual” OR “net produc*” OR bioturb* OR leaf OR litter OR “organic matter” OR decomposition OR metabol* OR “growth rate” OR “nutri* uptake” OR “nutri* cycling” OR carbon OR phosphor* OR nitrogen OR “uptake efficien*” OR “food web” OR “trophic interaction”) NOT (marin* OR pond OR coast* OR lake OR sea OR lent* OR marsh OR wetland).

We completed the primary systematic literature search on 30 September 2024. After this date, we screened newly published studies periodically and included them when they met the predefined eligibility criteria and provided relevant quantitative data for the meta‐analysis. This search identified 5911 potentially relevant publications. We screened studies in accordance with PRISMA guidelines (O'Dea et al. 2021) (Figure S1). We included studies that (1) evaluated the effects of hydromorphological restoration on ecosystem functions (Table 1), (2) were conducted in the field or experimental streamside channels, (3) employed a before‐after (BA), control‐impact (CI), or combined BACI study design, and (4) reported means, measures of variation, and sample sizes for restored and control sites.

**TABLE 1:** Summary of the dataset used in the meta‐analysis. *n* denotes the number of contributing studies and *k* the number of effect sizes. Ecosystem functions and biological‐structure attributes were grouped into comparable response categories, and restoration measures were assigned to four dominant groups based on their primary objective. Baseline reflects whether restored sites were compared to degraded or reference conditions.

	*n*	*k*	Description
Ecosystem functions (9 total)			
Gross primary production	24	104	Whole‐ecosystem oxygen production from photosynthesis
Ecosystem respiration	24	104	Whole‐ecosystem consumption of oxygen during degradation of organic matter
Denitrification	11	49	Nitrogen removal through microbial conversion of nitrate with organic carbon under anoxic conditions
NH_4_^+^ uptake velocity	9	50	Vertical velocity, measured at the stream reach scale, by which dissolved nutrients are removed from the water column and immobilised in particulate form or transformed into gaseous forms
NO_3_^−^ uptake velocity	9	45	
SRP uptake velocity	8	30	
OM decomposition	12	128	The rate at which OM loses mass due to leaching of soluble compounds, physical effects and the combined activities of microbes and invertebrates
Macroinvertebrate secondary production	10	42	Generation of new macroinvertebrate biomass over time
Sediment respiration	8	30	Oxygen consumption during the degradation of organic matter is measured within the stream bed
Biological‐structure attributes (19 total)			
Community abundance, biomass, richness, diversity	24	214	
Restoration measure (4 total)			
Instream habitat improvement	37	115	Direct manipulation of channel morphology, e.g., wood additions, channel reconfiguration
Riparian management	33	123	Vegetation‐driven bank & shading effects
Floodplain reconnection	22	66	Hydrologic connectivity & water retention
Barrier removal	18	61	Re‐establishing longitudinal connectivity
Baselines (3 total)			
Degraded vs. Reference	18	54	Difference of degraded relative to reference condition
Restored vs. Degraded	61	405	Difference of restored relative to degraded condition
Restored vs. Reference	27	123	Difference of restored relative to reference condition

For BACI studies, we decomposed the design into separate BA and CI contrasts, but retained only the CI contrasts for the main meta‐analysis. Accordingly, we excluded BA contrasts from both BA‐only and BACI studies because they quantify temporal change within the restoration site rather than differences between restored and contemporaneously sampled degraded or reference sites. Pooling BA and CI contrasts would therefore combine effect sizes with different ecological interpretations. Moreover, BA estimates cannot separate restoration effects from other temporal changes between sampling periods, which is particularly problematic for ecosystem functions that vary strongly with discharge, temperature, light, and resource availability. We therefore restricted the main analysis to CI contrasts to ensure a consistent effect‐size interpretation aligned with our aim of comparing restored streams with contemporaneous ecological benchmarks.

We screened studies using the metagear package (Lajeunesse 2016) in R (R Core Team 2024), and three researchers independently cross‐checked study inclusion. A total of 66 studies (582 effect sizes) were retained for data extraction and analysis (Haase et al. 2026). Within each study, we classified sampling sites as: (a) reference sites representing targeted ecological conditions, (b) degraded sites reflecting the state before restoration, and (c) restored sites where restoration measures had been implemented. Many studies reported multiple metrics for each ecosystem function (e.g., nutrient retention expressed as uptake length, uptake rate, or uptake velocity). To ensure comparability, we extracted one metric per function per study, favouring the most widely used or mechanistically representative measure (Table 1).

In several cases, ecosystem functions were measured across multiple restored sites and compared against a shared reference or a degraded site, without one‐to‐one pairing. This approach introduces statistical dependence among effect sizes originating from the same study, because multiple effect sizes share the same reference mean and associated sampling variance. We therefore explicitly modelled within‐study dependence in subsequent analyses using multilevel meta‐analysis (see Statistical Analysis below). Where measures of variation were missing, we imputed standard deviations from function‐specific variance distributions using the missing cases procedure (Nakagawa et al. 2023). We evaluated publication bias using Egger's regression test and model selection diagnostics in metafor (Viechtbauer 2010). The results showed no consistent evidence of publication bias from either approach for any function.

We recorded contextual and covariate information relevant to ecosystem recovery. For each study, we extracted: (1) biodiversity or community structure metrics when available (e.g., number of species, Shannon diversity, biomass, abundance); (2) the restoration measure, categorised into four groups: instream habitat improvement, riparian management, floodplain reconnection, and barrier removal; and (3) the restoration age, reported as the number of years between restoration implementation and measurement.

### 2.2 Statistical Analysis

We expressed effect sizes as log response ratios (logRR), with sampling variances calculated from reported means, standard deviations, and sample sizes. Because multiple effect sizes frequently originated from the same study (e.g., multiple functions or shared reference sites), we accounted for within‐study dependence by specifying a study‐level variance–covariance matrix using vcalc() in the R package metafor. This approach assigns a non‐zero covariance to effect sizes that share a reference mean, ensuring the appropriate propagation of uncertainty. We fitted all models as mixed‐effects meta‐regressions and based statistical inference on cluster‐robust variance estimators (CR2) implemented in the R package clubSandwich, which provides small‐sample‐adjusted standard errors and hypothesis tests for correlated effect sizes (Pustejovsky 2025).

We first analysed the full dataset of ecosystem‐function response ratios to test how functions respond to degradation (degraded vs. reference) and to restoration relative to the two baselines (restored vs. degraded; restored vs. reference). We also evaluated whether ecosystem‐function responses differed among restoration measures and included restoration measure as a moderator in the meta‐regression framework. To quantify temporal dynamics, we modelled effect sizes as a function of log‐transformed restoration age, calculated as log(1 + years since restoration), and derived slope estimates and contrasts from fitted models using linear combinations of fixed effects and CR2‐adjusted variance–covariance matrices.

Second, we assessed whether restoration‐induced changes in biological structure coincided with changes in ecosystem functioning and whether both components exhibited similar temporal trajectories following hydromorphological restoration. We conducted this analysis on a subset of studies that reported both biological‐structure attributes and functional metrics, for which we constructed structure–function pairs within each study. Biological‐structure attributes included taxonomic richness and diversity, abundance, biomass, and related standing‐stock metrics. Each pair consisted of one functional response variable and an ecologically related biological‐structure attribute (e.g., organic matter decomposition paired with macroinvertebrate richness; Table S1). To avoid drawing inferences from functions represented by very few independent studies, we restricted structure–function analyses to ecosystem functions represented by at least three structure–function pairs from at least three independent studies within each comparison design. Consequently, five functions were retained for restored–degraded comparisons and four for restored–reference comparisons. We first estimated the restoration response of biological structure within each function‐specific pairing. We then quantified structure–function contrasts as the difference between the paired functional and structural log response ratios (Function–Structure), such that negative values indicate that the functional logRR is more negative than the corresponding biological‐structure logRR, whereas positive values indicate that it is more positive. Multilevel meta‐regressions included response type, ecosystem‐function identity, and their interaction as moderators, with study and structure–function pair specified as nested random effects. Differences among function‐specific structure–function contrasts were evaluated using joint CR2‐adjusted Wald tests. We assessed temporal changes in structure–function contrasts by testing the interaction between response type and log‐transformed restoration age while accounting for ecosystem‐function identity. Function‐specific temporal contrasts were additionally estimated for functions with sufficient temporal replication. We based all structure–function estimates, confidence intervals, and hypothesis tests on CR2 robust variance estimation with small‐sample correction. To assess the influence of individual studies, we conducted leave‐one‐study‐out sensitivity analyses for function‐specific structure–function contrasts. We restricted formal sensitivity assessment to functions represented by at least four studies, ensuring at least three independent studies remained after omission.

## 3 Results

The final dataset included 281 streams or stream sites spanning first‐ to ninth‐order systems and elevations from 16 to 2974 m a.s.l. Studies spanned broad geographic and hydroclimatic gradients, ranging from approximately 19° N to 65° N latitude and from 123° W to 175° E longitude. They encompassed tropical, Mediterranean, temperate, and boreal biomes across North America, South America, Europe, Asia, and Oceania.

Ecosystem‐function responses varied substantially in magnitude and direction across degraded, restored, and reference conditions (Figure 1, Table S2). Degraded–reference comparisons provided the baseline for evaluating whether restoration shifted ecosystem functions back towards reference conditions. Among the nine functions, only gross primary production differed significantly between degraded and reference systems, with higher rates under degraded conditions (Table S2). Restoration did not consistently reverse degradation‐associated functional differences. In restored–degraded comparisons, only denitrification differed significantly, with lower rates in restored than in degraded systems (Table S2), whereas most other functions did not differ significantly between restored and degraded systems. Relative to reference conditions, nitrate uptake remained significantly lower in restored streams (Table S2). Gross primary production tended to be higher in restored than in reference streams, although the difference was not statistically significant (Table S2). Organic matter decomposition tended to be higher in restored than in reference streams, but this difference was not statistically significant (Table S2). Other ecosystem functions did not differ significantly between restored and reference systems.

**FIGURE 1:**
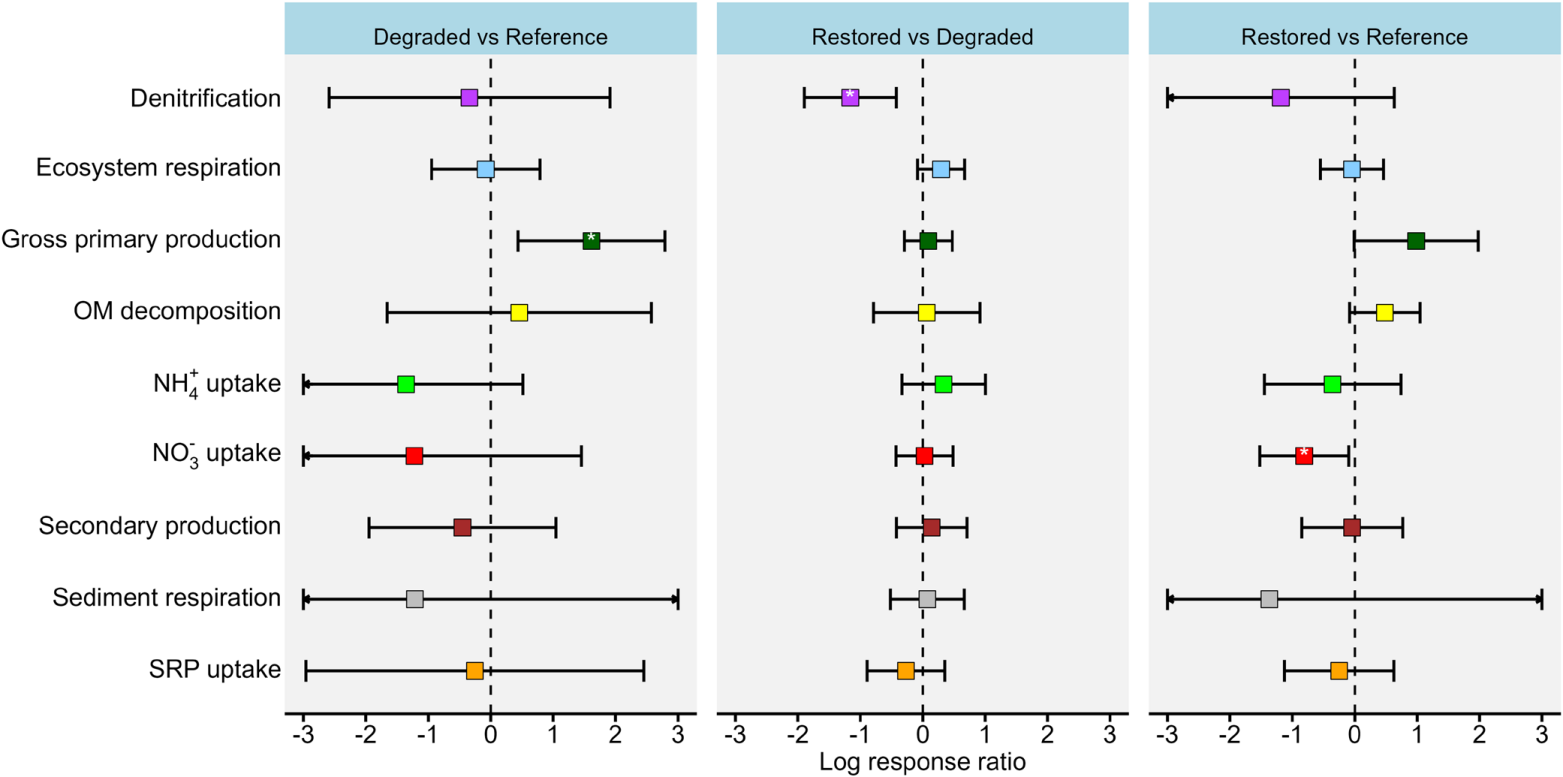
Ecosystem‐function responses across degraded, restored, and reference conditions. Squares show model‐estimated mean log response ratios (logRR) for degraded–reference, restored–degraded, and restored–reference comparisons from multilevel meta‐analytic models with CR2 robust inference; error bars indicate 95% confidence intervals. LogRR was calculated as the first condition divided by the second condition in each comparison, so positive values indicate higher functional rates in the first condition and negative values indicate higher rates in the second. Dashed vertical lines indicate no difference between comparison groups (logRR = 0). Confidence intervals extending beyond the plotted *x*‐axis range were truncated and are indicated by arrows. Asterisks denote estimates significantly different from zero.

We found no evidence that ecosystem‐function responses differed systematically among restoration measures after accounting for ecosystem function identity in either comparison baseline (restored–degraded: CR2 Wald test, *F*_3,3.49_ = 0.25, *p* = 0.860; restored–reference: *F*_3,8.01_ = 1.91, *p* = 0.206). We did not test interactions between functions and restoration measure because several function–measure combinations were underrepresented, resulting in redundant predictors and unstable coefficient estimates.

Temporal responses of ecosystem functioning varied by ecosystem function and comparison baseline (Table S3). In restored–degraded comparisons, denitrification and gross primary production declined significantly with restoration age, whereas most other ecosystem functions showed no significant temporal relationships. In restored–reference comparisons, temporal relationships were generally weak and inconsistent, and none of the function‐specific slopes differed significantly from zero. Thus, temporal changes relative to degraded conditions did not translate into a detectable convergence towards (or divergence from) reference conditions.

Before analysing structure–function contrasts, we first quantified the biological‐structure responses represented in the paired dataset. In restored–degraded comparisons, biological‐structure metrics showed positive mean responses, but only the biological metrics paired with gross primary production differed significantly from zero (Table S1). In restored–reference comparisons, none of the biological‐structure responses differed significantly from zero (Table S1).

Function–Structure contrasts were generally small and associated with substantial uncertainty (Table 2). None of the five restored–degraded or four restored–reference contrasts differed significantly from zero. Robust omnibus tests likewise provided no evidence that contrasts differed among ecosystem functions (restored–degraded: *F*_4,0.36_ = 0.63, *p* = 0.79; restored–reference: *F*_3,0.59_ = 0.91, *p* = 0.69), although the very low denominator degrees of freedom indicate limited effective information for these comparisons. Structure–function contrasts did not change systematically with restoration age for either comparison design (restored–degraded: *F*_1,4.73_ = 0.10, *p* = 0.77; restored–reference: *F*_1,3.84_ = 0.86, *p* = 0.41). Function‐specific temporal slopes varied in direction but were generally imprecise, with confidence intervals overlapping zero in all cases (Table S4). Leave‐one‐study‐out analyses (Table S5) showed that restored–degraded contrasts for gross primary production, organic matter decomposition, and secondary production, as well as the restored–reference contrast for organic matter decomposition, were stable in direction. Restored–reference estimates for gross primary production and particularly secondary production were more sensitive to individual studies (Table S5).

**TABLE 2:** Function‐specific structure–function contrasts across comparison designs. The Function–Structure contrast is defined as the ecosystem‐function log response ratio minus the corresponding biological‐structure log response ratio. Negative values therefore indicate that the ecosystem‐function response is more negative than the biological‐structure response, whereas positive values indicate that it is more positive. A value of zero indicates identical mean responses of ecosystem functioning and biological structure on the log response‐ratio scale. *k* is the number of paired structure–function observations and *n* is the number of independent studies contributing to the estimate. Only functions represented by at least three structure–function pairs from at least three independent studies were included. Confidence intervals and *p*‐values were derived using CR2 robust variance estimation with small‐sample correction.

	*k* pairs	*n* studies	Function−Structure contrast (±95% CI)	*p*
Restored vs. degraded				
Ecosystem respiration	7	3	0.27 (−0.22 to 0.77)	0.099
Gross primary production	9	4	−0.17 (−1.49 to 1.15)	0.523
NH_4_^+^ uptake	5	3	−0.68 (−4.88–3.53)	0.408
OM decomposition	86	7	−0.22 (−0.76 to 0.31)	0.269
Secondary production	44	8	−0.24 (−0.99 to 0.52)	0.220
Restored vs. reference				
Ecosystem respiration	7	3	−0.11 (−0.45 to 0.24)	0.180
Gross primary production	14	4	0.31 (−2.73 to 3.34)	0.573
OM decomposition	60	5	−0.18 (−0.62 to 0.26)	0.288
Secondary production	12	4	−0.98 (−4.04 to 2.07)	0.162

## 4 Discussion

Our synthesis revealed that hydromorphological restoration produces highly heterogeneous ecosystem‐function responses across restored stream ecosystems. Individual ecosystem functions varied substantially in magnitude and direction, and these responses depended on the ecological baseline used to evaluate restoration. Within the paired structure–function dataset, biological‐structure responses were consistently positive relative to degraded conditions, whereas corresponding functional responses showed no similarly consistent pattern. However, function‐specific structure–function contrasts were highly uncertain, and none differed significantly from zero. Together, these findings indicate that functional recovery cannot be reliably inferred from biological‐structure responses alone, and the limited independent replication for individual functions precludes strong conclusions about function‐specific mismatches.

The ecological interpretation of functional change critically depends on the baseline against which restoration is evaluated. Our degraded–reference comparison illustrates this particularly clearly for gross primary production. GPP was significantly higher in degraded than in reference streams, indicating that degradation had shifted this function above reference levels. Restored streams also tended to maintain higher GPP than reference streams, although this difference was not significant, suggesting that restoration did not fully reverse this deviation. Thus, an increase in a functional rate cannot necessarily be interpreted as ecological improvement. Denitrification provides a complementary example. Rates were significantly lower in restored than in degraded streams, potentially reflecting lower nutrient enrichment and organic matter accumulation or higher oxygen availability following restoration (Merill and Tonjes 2014), rather than a general loss of ecosystem functioning. Elevated denitrification increases nitrate removal via conversion to gaseous nitrogen, yet high denitrification rates do not necessarily indicate good ecological condition because they may be promoted by nitrate enrichment and organic matter accumulation characteristic of degraded systems (Mulholland et al. 2008). However, denitrification did not differ significantly between degraded and reference streams, so we could not determine whether the lower rates observed in restored reaches represent movement towards reference conditions. Similarly, for most other functions, degraded–reference contrasts were highly uncertain, limiting our ability to determine whether restoration‐associated changes represented movement towards or away from reference conditions. Nitrate uptake, for example, remained significantly lower in restored than in reference streams, whereas the corresponding degraded–reference contrast was too uncertain to establish whether this represented incomplete recovery from degradation. Restored–degraded contrasts therefore quantify intervention‐associated differences, whereas comparison with appropriate reference conditions is required to determine whether functional rates approach a restoration target.

Nevertheless, functional benchmarks for streams remain poorly developed. Gessner and Chauvet (2002) proposed reference‐based criteria for litter decomposition, and Young et al. (2008) extended this approach to ecosystem metabolism, including gross primary production and ecosystem respiration. Both frameworks emphasise that functional rates are strongly conditioned by natural environmental variation and that reference values therefore need to be interpreted within appropriate regional or stream‐type settings. Gessner and Chauvet (2002), for example, explicitly recognised that litter‐breakdown reference values vary among regions and stream types. Similarly, Young et al. (2008) showed marked differences in metabolism among reference streams with contrasting canopy conditions. These examples illustrate why universal functional thresholds are unlikely to transfer reliably across biomes and stream types. Existing large‐scale syntheses and emerging classifications of stream metabolic regimes (e.g., Bernhardt et al. 2018), along with coordinated experiments across biomes (e.g., Tiegs et al. 2019), offer promising routes to identify the environmental domains within which functional reference distributions may be defined. Such distributions could help distinguish statistically detectable deviations from ecologically meaningful impairment, although robust ecological thresholds may not exist for all processes. Until such thresholds are available, restoration assessment will require process‐specific, regionally contextualised definitions of desirable functional states rather than assuming that all functions should increase following restoration.

Heterogeneous responses across functions reflect differences in the environmental controls governing individual ecosystem processes. Hydromorphological restoration is frequently designed to improve channel structure, habitat heterogeneity, hydrologic connectivity, or biological communities, whereas ecosystem functions may additionally depend on catchment nutrient inputs, riparian conditions, organic matter supply, thermal regimes, sediment dynamics, and hydrologic residence time (Schinegger et al. 2012). Functional responses may therefore remain variable or shift in opposing directions when restoration modifies only part of the environmental template controlling a given process. At the broad level represented in our dataset, we found no evidence that ecosystem‐function responses differed systematically among restoration‐measure categories after accounting for ecosystem‐function identity. This lack of differentiation should not be interpreted as evidence that restoration design is irrelevant; rather, broad categories may be too coarse to capture whether an intervention modifies the specific environmental controls limiting a given ecosystem process. More broadly, functional recovery should not be expected as an automatic consequence of hydromorphological restoration, particularly when restoration actions and dominant process controls are only partially aligned (Kollmann et al. 2016; Verdonschot and Verdonschot 2023).

Temporal patterns reinforced this baseline dependence. Gross primary production and denitrification declined significantly with restoration age relative to degraded reaches, indicating that restoration‐associated functional differences can continue to change through time. However, none of the function‐specific restored–reference contrasts changed significantly with restoration age. Temporal change following restoration therefore did not translate into detectable convergence towards reference conditions. For GPP, the decline relative to degraded reaches is consistent with the possibility that elevated production shortly after restoration reflects transient increases in light availability or reduced riparian shading during early recovery (Kupilas et al. 2017), but the absence of a corresponding restored–reference trend cautions against interpreting this pattern as progressive functional recovery.

The paired analysis further highlights the distinction between biological and functional recovery. Biological‐structure responses relative to degraded conditions were positive across all retained function pairings, whereas corresponding functional responses were more variable. This pattern indicates that biological structure and ecosystem functioning are not necessarily changing in parallel following restoration. Biological‐structure metrics capture important aspects of ecological reassembly, including taxonomic richness and diversity, abundance, and biomass, yet ecosystem functions additionally depend on trophic structure, species interactions, biomass distribution, resource dynamics, and abiotic process controls that may recover along partly independent trajectories (Kollmann et al. 2016). However, given the considerable uncertainty in individual structure–function contrasts, these results should not be interpreted as evidence of a general decoupling between biological structure and ecosystem functioning. Moreover, the biological attributes available in the restoration literature encompassed not only taxonomic diversity measures but also community‐structure and standing‐stock attributes such as abundance, biomass, and biofilm‐related metrics. Consequently, our structure–function analysis does not represent a classical biodiversity–ecosystem functioning test, but instead evaluates whether restoration‐induced changes in biological structure and ecosystem functioning occur in parallel. Positive associations between biological and functional recovery would furthermore not necessarily imply direct causal coupling, because both may respond concurrently to the same restoration intervention (Bullock et al. 2011).

Asynchronous recovery across ecological dimensions is not unique to streams. Syntheses from wetlands, forests, and agroecosystems similarly show that biodiversity, ecosystem functions, and ecosystem services often recover at different rates and to different extents relative to reference conditions (Barral et al. 2015; Kimmell et al. 2023; Meli et al. 2014). Individual ecosystem processes may also follow distinct temporal trajectories during restoration (He et al. 2024). These findings reinforce the broader view that ecological recovery is multidimensional and process‐specific rather than characterised by uniform trajectories across ecosystem attributes.

This multidimensionality also means restoration success depends on the ecological attribute and management objective used to define it. Hydromorphological restoration may be judged successful when it improves biological quality elements targeted by regulatory frameworks. At the same time, the same intervention may produce little change in other communities, individual conservation targets, or ecosystem processes. Consequently, neither biological nor functional recovery constitutes a single universal endpoint. Restoration assessment should therefore define explicitly which ecosystem attributes are expected to respond and evaluate success using metrics and reference conditions aligned with those objectives. Functional targets likewise need to specify not only which processes are expected to recover, but also the direction and magnitude of desirable change relative to an appropriate ecological benchmark. Maximising all functional rates simultaneously is neither ecologically realistic nor necessarily desirable.

Stronger inference about functional recovery will also require study designs that distinguish restoration effects from background temporal variability while retaining ecologically meaningful benchmarks. Before–after comparisons quantify temporal change at restored sites but cannot by themselves separate restoration effects from concurrent changes in discharge, temperature, light, or resource availability. Contemporaneous degraded and reference comparisons provide ecological context, while BACI designs can combine temporal and spatial information and thereby strengthen attribution of observed changes to restoration. Future restoration studies should therefore prioritise replicated BACI‐type designs where feasible and explicitly define reference conditions appropriate to the ecosystem processes and management objectives being evaluated.

Together, our findings provide large‐scale empirical support for longstanding calls to integrate ecosystem functioning explicitly into restoration assessment and design (e.g., Naeem 2006; Palmer and Ruhi 2019). Hydromorphological restoration can alter ecosystem processes, but neither the direction nor magnitude of these changes provides a universal measure of ecological improvement. Functional recovery instead depends on the process considered, its environmental controls, the restoration target, and the baselines used to evaluate change. Advancing restoration ecology will therefore require monitoring frameworks that complement structural and biological indicators with direct measurements of ecosystem functioning, process‐specific and regionally appropriate reference conditions, and study designs that separate restoration effects from natural temporal variability.

## Author Contributions

**Ulrike Haase:** writing – original draft, methodology, writing – review and editing, data curation, visualization. **Daniel C. Allen:** conceptualization, writing – review and editing. **Christine Anlanger:** writing – review and editing. **Iola G. Boëchat:** writing – review and editing. **Clara Mendoza‐Lera:** writing – review and editing. **Wyatt F. Cross:** writing – review and editing. **Pia Ebeling:** formal analysis, software, writing – review and editing, data curation. **Davi G. Fernandes Cunha:** writing – review and editing. **Patrick Fink:** writing – review and editing, funding acquisition. **Nicolas Finkler:** writing – review and editing. **Björn Gücker:** conceptualization, writing – review and editing, funding acquisition. **Andreas Lorke:** writing – review and editing, funding acquisition. **Julia Pasqualini:** writing – review and editing, funding acquisition. **Christopher J. Patrick:** writing – review and editing. **Marc Peipoch:** writing – review and editing. **Oskar Schröder:** writing – review and editing, formal analysis, data curation, validation. **Ryan Sponseller:** writing – review and editing, funding acquisition. **Daniel von Schiller:** writing – review and editing, conceptualization, funding acquisition. **Markus Weitere:** writing – review and editing. **Mario Brauns:** conceptualization, writing – original draft, methodology, writing – review and editing, formal analysis, supervision, data curation, visualization, funding acquisition.

## Funding

B.G. was funded by Fundação de Amparo à Pesquisa do Estado de Minas Gerais (FAPEMIG APQ‐06893‐24) and Conselho Nacional de Desenvolvimento Científico e Tecnológico (CNPq #310551/2022‐6). J.P., C.A., P.F., A.L., C.M.L., M.W., and M.B. were funded through the 2020‐2021 Biodiversa+ and Water JPI joint call for research projects under the BiodivRestore ERA‐NET Cofund (GA N°101003777), with the EU and the Federal Ministry of Education and Research, Germany (Grant 16LW0174K, 16LW0175). DvS was funded through the 2020‐2021 Biodiversa+ and Water JPI joint call for research projects, under the BiodivRestore ERA‐NET Cofund (GA N°101003777), with Grant PCI2022‐132930 funded by MCIU/AEI/10.13039/501100011033 and by the European Union NextGeneration EU/PRTR. DvS is a Serra Húnter Fellow.

## Conflicts of Interest

The authors declare no conflicts of interest.

## Supporting information


**Figure S1:** PRISMA 2020 flow diagram of the total number of studies identified, screened and included in the meta‐analysis.
**Table S1:** Biological‐structure responses within the paired structure–function dataset. Biological‐structure effect sizes are grouped according to the ecosystem function with which they were paired in the respective studies. Listed metrics indicate the biological attributes contributing to each function group. Biological‐structure responses are expressed as log response ratios (logRR), calculated as the biological‐structure value in restored streams divided by that in degraded or reference streams, as shown in the comparison. Thus, positive logRR values indicate higher biological‐structure metric values in restored streams, whereas negative values indicate lower values. A logRR of zero indicates no difference between conditions. *k* is the number of paired structure–function observations and n is the number of independent studies contributing to the estimate. Only function groups represented by at least three structure–function pairs from at least three independent studies were included. Confidence intervals and *p*‐values were derived using CR2 robust variance estimation with small‐sample correction.
**Table S2:** Multilevel meta‐analysis of ecosystem‐function responses across degraded–reference, restored–degraded, and restored–reference comparisons. Effect sizes are expressed as log response ratios (logRR), calculated as the functional rate in the first condition divided by that in the second condition named in each comparison. Positive logRR values indicate higher functional rates in the first condition, whereas negative values indicate higher rates in the second condition; a logRR of zero indicates no difference between conditions. Confidence intervals including zero indicate that the two conditions do not differ significantly. Degraded–reference comparisons provide the ecological baseline needed to interpret whether restored–degraded or restored‐reference differences represent movement towards or away from reference conditions; increases or decreases in functional rates do not inherently indicate ecological recovery.
**Table S3:** Function‐specific temporal trends in ecosystem‐function responses for restored–degraded and restored–reference comparisons. Temporal slopes describe the change in the function‐specific log response ratio (logRR) with increasing log‐transformed restoration age, calculated as log(1 + years since restoration). Positive slopes indicate that the logRR becomes more positive with restoration age, whereas negative slopes indicate that it becomes more negative; a slope of zero indicates no systematic temporal change. Because logRR is calculated as the first condition divided by the second condition named in each comparison, the ecological interpretation of a positive or negative slope depends on the comparison considered and does not inherently indicate functional recovery. Confidence intervals including zero indicate no statistically detectable temporal trend.
**Table S4:** Function‐specific temporal trends in structure–function contrasts. The Function − Structure contrast represents the difference between the ecosystem‐function log response ratio and the corresponding biological‐structure log response ratio. Temporal slopes describe how this contrast changes with increasing log‐transformed restoration age, calculated as log(1 + years since restoration). Positive slopes indicate that functional responses become more positive relative to biological‐structure responses over time, whereas negative slopes indicate that functional responses become more negative relative to biological‐structure responses; a slope of zero indicates no systematic temporal change in the structure–function contrast. *k* is the number of paired structure–function observations, *n* studies the number of independent studies, and *n* time points the number of distinct restoration ages contributing to the estimate. Functions first had to satisfy the main inclusion criterion of at least three structure–function pairs from at least three independent studies; temporal models additionally required both response types across at least two studies and two distinct time values. Confidence intervals including zero indicate no statistically detectable temporal trend.
**Table S5:** Leave‐one‐study‐out sensitivity analysis of function‐specific structure–function contrasts. The Function−Structure contrast is defined as the ecosystem‐function log response ratio minus the corresponding biological‐structure log response ratio. Negative values therefore indicate that the ecosystem‐function response is more negative than the biological‐structure response, whereas positive values indicate that it is more positive. The full‐model contrast is the estimate obtained from the complete structure–function dataset. The leave‐one‐study‐out (LOO) range gives the minimum and maximum contrast obtained after sequentially removing each contributing study. Maximum absolute change from the full model is the largest absolute difference between a LOO estimate and the full‐model contrast. Sign change indicates whether omission of at least one study reversed the direction of the estimated Function–Structure contrast. Formal LOO analyses were restricted to functions represented by at least four independent studies, ensuring that at least three studies remained after omission. We evaluated functions represented by only three studies diagnostically but did not include them in this table.

## Data Availability

The data and all original sources that support the findings of this study are openly available in Dryad (https://doi.org/10.5061/dryad.m37pvmdjq) and in the Supporting Information of this article.
