# Supplementary material for: Hydromorphological Restoration of Streams Does Not Ensure the Recovery of Ecosystem Functioning
Source: Glob Chang Biol. 2026 Oct 22;32(10):e71126. doi: 10.1111/gcb.71126 (PMC13688068; doi:10.1111/gcb.71126)
Supplement: Supplementary file 1 [file GCB-32-e71126-s001.docx]

Supplementary Information

**Hydromorphological restoration of streams does not ensure the recovery of ecosystem functioning**

Ulrike Haase, Daniel C. Allen, Christine Anlanger, Iola G. Boëchat, Clara Mendoza-Lera, Wyatt F. Cross, Pia Ebeling, Davi G. Fernandes Cunha, Patrick Fink, Nicolas Finkler, Björn Gücker, Andreas Lorke, Julia Pasqualini, Christopher J. Patrick, Marc Peipoch, Oskar Schröder, Ryan Sponseller, Daniel von Schiller, Markus Weitere, Mario Brauns

**Table S1. Biological-structure responses within the paired structure–function dataset**. Biological-structure effect sizes are grouped according to the ecosystem function with which they were paired in the respective studies. Listed metrics indicate the biological attributes contributing to each function group. Biological-structure responses are expressed as log response ratios (logRR), calculated as the biological-structure value in restored streams divided by that in degraded or reference streams, as shown in the comparison. Thus, positive logRR values indicate higher biological-structure metric values in restored streams, whereas negative values indicate lower values. A logRR of zero indicates no difference between conditions. k is the number of paired structure–function observations and n is the number of independent studies contributing to the estimate. Only function groups represented by at least three structure–function pairs from at least three independent studies were included. Confidence intervals and p-values were derived using CR2 robust variance estimation with small-sample correction.

| **Function paired with biological structure** | **Biological-structure metrics** | **k** | **n** | **logRR**  **(± 95% CI)** | **p** |  |
| --- | --- | --- | --- | --- | --- | --- |
| **Restored vs Degraded** | | | | | | |
| Ecosystem respiration | Bacterial abundance, Bacterial cell biomass, Biofilm biomass, BOM biomass, OM biomass | 7 | 3 | 0.34  (-0.23 - 0.92) | 0.157 |  |
| Gross primary production | Biofilm biomass, Biofilm chlorophyll-a, BOM biomass, Submerged macrophyte cover, Submerged macrophyte richness | 9 | 4 | 0.83  (0.22 - 1.45) | 0.021 |  |
| OM decomposition | Bacterial diversity, Bacterial richness, Detritivore abundance, Macroinvertebrate abundance, Macroinvertebrate biomass, Macroinvertebrate diversity, Macroinvertebrate evenness, Macroinvertebrate richness, Shredder abundance, Shredder biomass, Shredder evenness, Shredder richness | 86 | 7 | 0.12  (-0.26 - 0.50) | 0.472 |  |
| Secondary production | Macroinvertebrate abundance, Macroinvertebrate biomass, Macroinvertebrate diversity, Macroinvertebrate evenness, Macroinvertebrate richness | 44 | 8 | 0.30  (-0.31 - 0.91) | 0.284 |  |
| NH₄⁺ uptake | Biofilm chlorophyll-a | 5 | 3 | 1.27  (-0.38 - 2.92) | 0.082 |  |
| **Restored vs Reference** | | | | | | |
| Ecosystem respiration | Biofilm biomass, Cyanobacteria biomass, Diatom biomass | 7 | 3 | 0.30  (-0.45 - 1.05) | 0.332 |  |
| Gross primary production | Biofilm biomass, Biofilm chlorophyll-a, Cyanobacteria biomass, Diatom biomass, Submerged macrophyte cover, Submerged macrophyte richness | 14 | 4 | 0.19  (-0.30 - 0.67) | 0.345 |  |
| OM decomposition | Bacterial diversity, Bacterial richness, Detritivore abundance, Fungal diversity, Fungal richness, Macroinvertebrate abundance, Macroinvertebrate diversity, Macroinvertebrate evenness, Macroinvertebrate richness, Shredder abundance, Shredder biomass, Shredder evenness, Shredder richness | 60 | 5 | -0.08  (-0.59 - 0.43) | 0.709 |  |
| Secondary production | Macroinvertebrate abundance, Macroinvertebrate biomass, Macroinvertebrate diversity, Macroinvertebrate evenness, Macroinvertebrate richness | 12 | 4 | 0.51  (-0.74 - 1.75) | 0.278 |  |

**Table S2. Multilevel meta-analysis of ecosystem-function responses across degraded–reference, restored–degraded, and restored–reference comparisons**. Effect sizes are expressed as log response ratios (logRR), calculated as the functional rate in the first condition divided by that in the second condition named in each comparison. Positive logRR values indicate higher functional rates in the first condition, whereas negative values indicate higher rates in the second condition; a logRR of zero indicates no difference between conditions. Confidence intervals including zero indicate that the two conditions do not differ significantly. Degraded–reference comparisons provide the ecological baseline needed to interpret whether restored–degraded differences represent movement towards or away from reference conditions; increases or decreases in functional rates do not inherently indicate ecological recovery.

|  | Function | logRR | 95% CI | p |
| --- | --- | --- | --- | --- |
| **Degraded–Reference** | Denitrification | -0.34 | -2.59 - 1.91 | 0.673 |
|  | Ecosystem respiration | -0.08 | -0.95 - 0.79 | 0.839 |
|  | Gross primary production | 1.61 | 0.44 - 2.79 | 0.013 |
|  | NH₄⁺ uptake | -1.36 | -3.23 - 0.52 | 0.111 |
|  | NO₃⁻ uptake | -1.22 | -3.9 - 1.45 | 0.139 |
|  | OM decomposition | 0.46 | -1.66 - 2.57 | 0.545 |
|  | SRP uptake | -0.25 | -2.96 - 2.45 | 0.789 |
|  | Secondary production | -0.45 | -1.95 - 1.04 | 0.386 |
|  | Sediment respiration | -1.22 | -5.96 - 3.53 | 0.215 |
| **Restored–Degraded** | Denitrification | -1.16 | -1.90 - -0.42 | 0.007 |
|  | Ecosystem respiration | 0.29 | -0.08 - 0.67 | 0.120 |
|  | Gross primary production | 0.09 | -0.30 - 0.47 | 0.634 |
|  | NH₄⁺ uptake | 0.34 | -0.33 – 1.00 | 0.291 |
|  | NO₃⁻ uptake | 0.03 | -0.43 - 0.49 | 0.884 |
|  | OM decomposition | 0.06 | -0.79 - 0.92 | 0.857 |
|  | SRP uptake | -0.27 | -0.89 - 0.35 | 0.353 |
|  | Secondary production | 0.14 | -0.42 - 0.71 | 0.565 |
|  | Sediment respiration | 0.07 | -0.52 - 0.66 | 0.780 |
| **Restored–Reference** | Denitrification | -1.19 | -3.00 - 0.63 | 0.126 |
|  | Ecosystem respiration | -0.04 | -0.55 - 0.46 | 0.859 |
|  | Gross primary production | 0.98 | -0.01 - 1.98 | 0.052 |
|  | NH₄⁺ uptake | -0.36 | -1.45 - 0.74 | 0.405 |
|  | NO₃⁻ uptake | -0.81 | -1.52 - -0.10 | 0.034 |
|  | OM decomposition | 0.48 | -0.08 - 1.04 | 0.090 |
|  | SRP uptake | -0.25 | -1.13 - 0.63 | 0.485 |
|  | Secondary production | -0.04 | -0.85 - 0.77 | 0.881 |
|  | Sediment respiration | -1.37 | -6.55 - 3.81 | 0.214 |

**Table S3. Function-specific temporal trends in ecosystem-function responses for restored–degraded and restored–reference comparisons**. Temporal slopes describe the change in the function-specific log response ratio (logRR) with increasing log-transformed restoration age, calculated as log(1 + years since restoration). Positive slopes indicate that the logRR becomes more positive with restoration age, whereas negative slopes indicate that it becomes more negative; a slope of zero indicates no systematic temporal change. Because logRR is calculated as the first condition divided by the second condition named in each comparison, the ecological interpretation of a positive or negative slope depends on the comparison considered and does not inherently indicate functional recovery. Confidence intervals including zero indicate no statistically detectable temporal trend.

| Function | Temporal slope of logRR | SE | 95% CI | p | |
| --- | --- | --- | --- | --- | --- |
| **Restored–degraded** |  |  |  |  | |
| Denitrification | -0.40 | 0.15 | -0.79 - -0.01 | 0.047 | |
| Ecosystem respiration | -0.01 | 0.16 | -0.93 - 0.9 | 0.938 | |
| Gross primary production | -0.20 | 0.02 | -0.34 - -0.05 | 0.032 | |
| NH₄⁺ uptake | 0.20 | 0.24 | -0.54 - 0.95 | 0.465 | |
| NO₃⁻ uptake | 0.41 | 0.17 | -0.13 - 0.95 | 0.095 | |
| OM decomposition | 0.05 | 0.02 | -0.17 - 0.27 | 0.227 | |
| SRP uptake | -0.05 | 0.22 | -0.77 - 0.67 | 0.841 | |
| Secondary production | -0.23 | 0.20 | -2.2 - 1.74 | 0.446 | |
| Sediment respiration | 0.27 | 0.20 | -0.5 - 1.03 | 0.296 | |
| **Restored–reference** |  |  |  |  |  |
| Denitrification | -0.44 | 0.32 | -2.66 - 1.77 | 0.346 |  |
| Ecosystem respiration | -0.27 | 0.16 | -1.67 - 1.13 | 0.319 |  |
| Gross primary production | 0.42 | 0.09 | -0.22 - 1.05 | 0.087 |  |
| NH₄⁺ uptake | 0.31 | 0.24 | -1.98 - 2.6 | 0.398 |  |
| NO₃⁻ uptake | -0.43 | 0.37 | -2.71 - 1.84 | 0.401 |  |
| OM decomposition | 0.00 | 0.10 | -1.19 - 1.19 | 0.985 |  |
| SRP uptake | -0.32 | 1.00 | -3.97 - 3.33 | 0.776 |  |
| Secondary production | 0.00 | 0.48 | -5.02 - 5.02 | 0.997 |  |
| Sediment respiration | 1.26 | 2.57 | -10.51 - 13.03 | 0.675 |  |

**Table S4. Function-specific temporal trends in structure–function contrasts**. The Function − Structure contrast represents the difference between the ecosystem-function log response ratio and the corresponding biological-structure log response ratio. Temporal slopes describe how this contrast changes with increasing log-transformed restoration age, calculated as log(1 + years since restoration). Positive slopes indicate that functional responses become more positive relative to biological-structure responses over time, whereas negative slopes indicate that functional responses become more negative relative to biological-structure responses; a slope of zero indicates no systematic temporal change in the structure–function contrast. k is the number of paired structure–function observations, n studies the number of independent studies, and n time points the number of distinct restoration ages contributing to the estimate. Functions first had to satisfy the main inclusion criterion of at least three structure–function pairs from at least three independent studies; temporal models additionally required both response types across at least two studies and two distinct time values. Confidence intervals including zero indicate no statistically detectable temporal trend.

| Function | k | n studies | n time points | Temporal slope of Function − Structure contrast | SE | 95% CI | p |
| --- | --- | --- | --- | --- | --- | --- | --- |
| **Restored vs Degraded** |  |  |  |  |  |  |  |
| Ecosystem respiration | 7 | 3 | 2 | 0.10 | 0.63 | -5.23 - 5.42 | 0.900 |
| Gross primary production | 9 | 4 | 3 | 1.06 | 0.34 | -2.89 - 5.01 | 0.189 |
| OM decomposition | 86 | 7 | 11 | -0.14 | 0.21 | -1.04 - 0.76 | 0.576 |
| Secondary production | 44 | 8 | 6 | -0.42 | 0.22 | -1.23 - 0.39 | 0.174 |
| NH₄⁺ uptake | 5 | 3 | 3 | 2.95 | 3.72 | -43.72 - 49.62 | 0.572 |
| **Restored vs Reference** |  |  |  |  |  |  |  |
| Ecosystem respiration | 7 | 3 | 3 | -0.07 | 0.20 | -2.12 - 1.97 | 0.776 |
| Gross primary production | 14 | 4 | 4 | 0.74 | 0.32 | -2.57 - 4.04 | 0.242 |
| OM decomposition | 60 | 5 | 7 | -0.02 | 0.17 | -1.03 - 0.99 | 0.921 |
| Secondary production | 12 | 4 | 3 | -2.07 | 0.89 | -11.61 - 7.47 | 0.245 |

**Table S5. Leave-one-study-out sensitivity analysis of function-specific structure–function contrasts**. The Function − Structure contrast is defined as the ecosystem-function log response ratio minus the corresponding biological-structure log response ratio. Negative values therefore indicate that the ecosystem-function response is more negative than the biological-structure response, whereas positive values indicate that it is more positive. The full-model contrast is the estimate obtained from the complete structure–function dataset. The leave-one-study-out (LOO) range gives the minimum and maximum contrast obtained after sequentially removing each contributing study. Maximum absolute change from the full model is the largest absolute difference between a LOO estimate and the full-model contrast. Sign change indicates whether omission of at least one study reversed the direction of the estimated Function-Structure contrast. Formal LOO analyses were restricted to functions represented by at least four independent studies, ensuring that at least three studies remained after omission. We evaluated functions represented by only three studies diagnostically but did not include them in this table.

| Function | n | Full-model contrast | LOO  range | Maximum absolute change from full model | Sign  change |  |
| --- | --- | --- | --- | --- | --- | --- |
| **Restored vs Degraded** | | | | | | |
| Gross primary production | 4 | -0.17 | -0.46 - -0.07 | 0.29 | No |  |
| OM decomposition | 7 | -0.22 | -0.37 - -0.13 | 0.15 | No |  |
| Secondary production | 8 | -0.24 | -0.32 - -0.13 | 0.11 | No |  |
| **Restored vs Reference** |  |  |  |  |  |  |
| Gross primary production | 4 | 0.31 | -0.01 - 0.84 | 0.53 | Yes |  |
| OM decomposition | 5 | -0.18 | -0.30 - -0.10 | 0.11 | No |  |
| Secondary production | 4 | -0.98 | -1.02 - 0.17 | 1.15 | Yes |  |

Figure S1: PRISMA 2020 flow diagram of the total number of studies identified, screened and included in the meta-analysis
